# Multi-Compartment 3D-Cultured Organ-on-a-Chip: Towards a Biomimetic Lymph Node for Drug Development

**DOI:** 10.3390/pharmaceutics12050464

**Published:** 2020-05-19

**Authors:** Aya Shanti, Bisan Samara, Amal Abdullah, Nicholas Hallfors, Dino Accoto, Jiranuwat Sapudom, Aseel Alatoom, Jeremy Teo, Serena Danti, Cesare Stefanini

**Affiliations:** 1 Healthcare Engineering Innovation Center, Biomedical Engineering Department, Khalifa University of Science and Technology, Abu Dhabi P.O. Box 127788, UAE; aya.shanti@ku.ac.ae (A.S.); bisansamara@gmail.com (B.S.); amal-shukri@hotmail.com (A.A.); nicholas.hallfors@ku.ac.ae (N.H.); 2 School of Mechanical & Aerospace Engineering, Nanyang Technological University, 50 Nanyang Avenue, Singapore 639798, Singapore; daccoto@ntu.edu.sg; 3 Division of Engineering, New York University Abu Dhabi, Abu Dhabi P.O. Box 129188, UAE; jiranuwat.sapudom@nyu.edu (J.S.); aseel.alatoom@nyu.edu (A.A.); jeremy.teo@nyu.edu (J.T.); 4 Department of Biomedical and Mechanical Engineering, New York University, P.O. Box 903, New York, NY 10276-0903, USA; 5 Department of Civil and Industrial Engineering, University of Pisa, 56122 Pisa, Italy; serena.danti@unipi.it

**Keywords:** biomimicry, drug development, lymph node, microfabrication, microfluidics, organ-on-a-chip

## Abstract

The interaction of immune cells with drugs and/or with other cell types should be mechanistically investigated in order to reduce attrition of new drug development. However, they are currently only limited technologies that address this need. In our work, we developed initial but significant building blocks that enable such immune-drug studies. We developed a novel microfluidic platform replicating the Lymph Node (LN) microenvironment called LN-on-a-chip, starting from design all the way to microfabrication, characterization and validation in terms of architectural features, fluidics, cytocompatibility, and usability. To prove the biomimetics of this microenvironment, we inserted different immune cell types in a microfluidic device, which showed an in-vivo-like spatial distribution. We demonstrated that the developed LN-on-a-chip incorporates key features of the native human LN, namely, (i) similarity in extracellular matrix composition, morphology, porosity, stiffness, and permeability, (ii) compartmentalization of immune cells within distinct structural domains, (iii) replication of the lymphatic fluid flow pattern, (iv) viability of encapsulated cells in collagen over the typical timeframe of immunotoxicity experiments, and (v) interaction among different cell types across chamber boundaries. Further studies with this platform may assess the immune cell function as a step forward to disclose the effects of pharmaceutics to downstream immunology in more physiologically relevant microenvironments.

## 1. Introduction

Every year, new drug candidates, which previously underwent in vitro and in silico investigations, are proposed to enter preclinical and clinical studies [1,2,3]. Although analytical and experimental methodologies have enhanced our capability to fine-tune drug metabolism and pharmacokinetic profiles, failures of efficacy and safety is still observed. Drug toxicity alone has contributed to 1/3 of failures of drug candidates and the immune system has been invoked in playing a key role in drug toxicity [4]. All pharmaceutical drugs interact with the immune system, but the actual mechanism and immunological outcome of such interaction is not well-defined [3]. Not understanding the immunological–pharmaceutical interactions hides prospective side effects, hinders drug development, and limits discovery. As a consequence, the effect of a drug candidate on the immune system, known as immunotoxicity, is often underestimated along the drug development process. In addition, the techniques currently available to evaluate the immunotoxicity of drug candidates during preclinical stages often lack reliability and sensitivity, thus resulting in many drugs, which have initially passed the preclinical phase of drug approval, to fail in the clinical phase. This ultimately represents a significant waste of time, resources, and money [4,5,6,7].

The latest revolution in the pharmaceutical industry is the use of immune cells for cell-based drug delivery [8,9,10], as it is superior compared to many other delivery systems and has broader applicability [9,11]. Further evaluation needs to be performed on such modified immune cells, as the introduction of cell carriers to deliver drugs could also interrupt natural physiological conditions or exacerbate already diseased tissues.

Due to the profound interaction between drugs and immune cells, novel bioengineered platforms could be investigated with the intent of enabling a more proficient and less toxic drug design.

Studies on cellular systems should be performed using experimental setups that closely mimic the native microenvironment in which the cells can perform a physiological-like function. Such setups are made possible by the microfluidic technology, which is widely employed in biological research, as it allows miniaturization of components, efficient consumption of reagents, maximal output from precious samples, and high-throughput analysis of cellular behavior and dynamics in their microenvironment [12,13,14]. Harnessing of the microfluidic technology has resulted in many cellular and tissue systems being mimicked and miniaturized in research labs and in more established models now being commercially available [15,16,17,18]. However, in spite of the large number of models proposed for drug development applications, including those of the skin, liver, and gut, the recreation of the complex architecture of the lymph node (LN) has not been provided yet [13,19]. Only recently has the need for developing an LN-on-a-chip been recognized, as the immune outcome from LN is a key determinant of not only the immunotoxicity of drugs, but also the response to viruses, bacteria, and other foreign particles [20].

**Figure 1:**
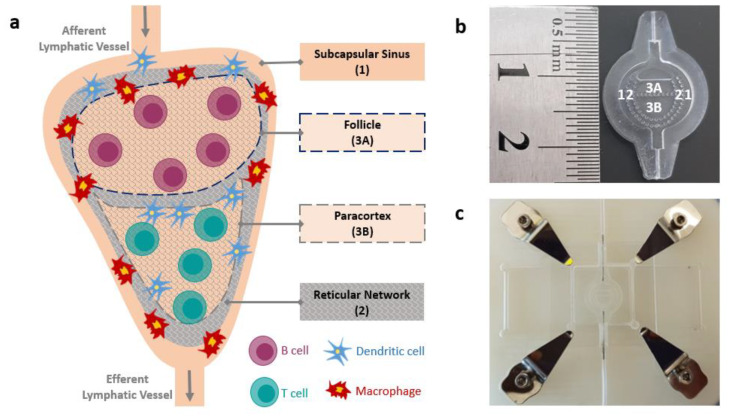
Artificial biomimetic LN-on-a-chip. (**a**) Schematic of the human LN showing the key anatomical features. (**b**) LN-on-a-chip designed to mimic the human LN. Region labelled 1 resembles the subcapsular sinus, region labelled 2 resembles the reticular network, region labelled 3A resembles the follicle, and region labelled 3B resembles the paracortex. Regions 3A and 3B are the cellular regions loaded with immune cells embedded in 3D hydrogel matrices. (**c**) The clamping system used to seal the device and produce a functional system that can be loaded with immune cells.

The LN is a secondary lymphoid organ that provides a unique cellular environment for immune cells (Figure 1a). It collects and filters the draining interstitial fluid that flows across tissues known as lymph before it is eventually returned back into active blood circulation [21,22]. The LN is the site whereby the body invokes immune responses towards toxic agents via triggering activation, proliferation, and differentiation of lymphocytes [13]. To facilitate optimal lymphocyte activation, the LN provides a unique structural microenvironment. It can be best described as a densely packed cellular environment with an extracellular matrix (ECM) primarily made up of type I collagen [23,24]. The LN is compartmentalized into distinct cellular microdomains, which are populated by either B cells or T cells, as shown in Figure 1a [25,26]. When the lymph enters an LN through afferent vessels, it first arrives at the subcapsular sinus (SCS), which is a hollow space below the capsule that covers an LN [27]. The SCS is characterized by the presence of macrophages [27]. As lymph flows through the SCS, a fraction of the fluid gets diverted laterally into a web-like conduit system formed by reticular collagen fibrils that penetrate the B cell follicle as well as the paracortex [28]. The conduit system plays a crucial role in delivering antigens and antigen-presenting cells [29,30,31].

The flow rate and pressure of the lymphatic flow within the LN are also believed to be key modulators of adaptive immunity [28,30,32]. Values of lymph flow rate vary in literature. However, on average, the homeostatic lymph speed measured in hand-to-axillary lymphatics of healthy subjects, is estimated to be around 1 mm/s, which could increase in case of LN inflammation and decrease under some pathologies, such as lymphedema [28,33].

A biomimetic LN should recreate the structural microenvironment of native LN and should incorporate its key functional features, primarily, the confinement of different cellular communities within distinct anatomical microdomains and the sustenance of a unique flow pattern, in which the flow is primarily present in the peripheral regions. Furthermore, such biomimetic LN should facilitate studies aimed at quantifying immune cell–cell interactions and cell–antigen interactions.

Microfluidic devices have emerged as potential means for recreating important aspects of cellular microenvironments in vitro by monitoring cell responses in real time, can benefit from microfabrication techniques, which have been shown to allow production of high-fidelity intricate structures to reproduce the biological scaffold [34,35,36].

This study aims at developing an in vitro model of the LN to closely mimic the native LN microenvironment, ultimately enabling immunological quantification studies. We used microfabrication techniques to obtain a microfluidic system. We then encapsulated different immune cell types in 3D biomimetic matrices and accurately introduced them into the device. We finally showed that cellular residents occupy distinct domains within the developed artificial LN and that the fluid flow within the system is comparable to the flow of lymphatic fluid through the native LN. Furthermore, we demonstrated interaction between different cell types across chamber boundaries and demonstrated long-term cell viability and activity enhanced by perfusion. We additionally designed and manufactured a biomimetic LN-on-a-chip, transparent to support real-time immunological studies.

This LN-on-a-chip opens up the possibility to characterize immune cells and identify behavioral clusters under several states, such as unperturbed states, activated states, and when immune cells are exposed to antigens or different pharmaceutical drugs. Investigations into the mechanisms of interaction between the immune system and drug candidates are lacking and, if available, could unlock significant knowledge to cut down the high cost of drug development as well as to reduce the high attrition rate.

## 2. Materials and Methods

### 2.1. LN-on-a-Chip Design and Fabrication

The developed LN microenvironment model is a multi-compartmentalized bioreactor consisting of an elliptical body, an inlet, and an outlet aperture (Figure 1b). The elliptical body further consists of an outermost region (region 1), a middle region (region 2), and an inner region (region 3), and each region is separated from the adjacent one by circularly distributed micropillars. The inner region is in turn split into two regions: a top region (region 3A) and a bottom region (region 3B) centrally located within the device. The two regions are separated by a horizontal row of micropillars and filled with distinct types of immune cells embedded in 3D hydrogels. Micropillars have been shown to allow confinement of different ECM components within distinct regions [37]. Furthermore, the microfluidic device is designed to mimic the in vivo LN architecture as each region within the device corresponds to an LN component as follows: region 1 corresponds to the subcapsular sinus region, region 2 corresponds to the reticular conduit network which mediates the transport of antigens from the peripheral SCS into the interior of the LN, region 3A corresponds to the follicle where B lymphocytes reside, and region 3B corresponds to the paracortex where T lymphocytes reside.

The microfluidic LN-on-a-chip was 3D-modelled using the AutoCAD 2016 software (Autodesk Inc., Europe). The major and minor axes of the elliptical body of the device have the dimensions of 10.2 mm and 10.0 mm, respectively. The radius of all micropillars is 225 µm and their height is 1500 µm. The spacing between each micropillar and the next is 180 µm. The inlet and outlet apertures are 2.0 mm by 1.3 mm each. These dimensional features are defined in accordance with those of the native LN [38,39].

The microfluidic LN-on-a-chip was fabricated by micromolding from polydimethylsiloxane (PDMS) (SYLGARD 184, Dow Corning, MI, USA). A master mold made of polytetrafluoroethylene (PTFE) was first manufactured using a computer numerical controlled micro-milling machine (CN-MAX 80-45, TEA Technology Engineering Automation SRL, Pisa, Italy). Next, a PDMS base-curing agent mixture (10:1 ratio) was prepared according to the manufacturer’s recommendation and poured into the mold. The PDMS-filled mold was degassed in a vacuum chamber for 30 min and then kept in ambient air for 24 h. This ensures that all bubbles trapped in the mixture, which could compromise the overall strength of the microfluidic device and impair development of the micropillars, are eliminated before PDMS cures. The mold was finally heated at 70 °C for 2 h and the cured PDMS device was peeled off from the mold.

Prior to loading, the PDMS device was treated with O2 plasma (100 W, 2.25 Torr, 5 min) to convert its surface from hydrophobic to hydrophilic as necessary to support adhesion of hydrogels and flow of media. After oxygen plasma treatment, cellular components embedded in hydrogels were loaded into regions 3A and 3B of the device using a micropipette (Eppendorf, Hamburg, Germany). The device was then sealed using a custom mechanical clamping system such that the microfluidic device was sandwiched between two glass slides, which were in turn tightened using sterile screws to create a perfect seal. The clamped device is shown in Figure 1c.

### 2.2. Hydrogel Preparation

Collagen matrices were reconstituted following an established protocol [40,41]. Briefly, type I collagen (Advanced Biomatrix, Carlsbad, CA, USA) was mixed with 250 mM phosphate buffer at pH 7.5 to achieve a final collagen concentration of 2 mg/mL. To initiate fibrillogenesis, the prepared collagen solution was incubated at 37 °C, 5% CO_2_, and 95% humidity for 30 min. The resultant collagen matrices were characterized to ensure that immune cells are placed in an environment comparable to that of the native LN. Specifically, collagen matrices were employed in topological analysis, matrix permeability analysis, and flow analysis. The parameters obtained from these analyses were also utilized to run a computational simulation of the developed microfluidic LN.

### 2.3. Hydrogel Characterization

#### 2.3.1. Topological Analysis

For topological analysis, the prepared 3D collagen matrices were transferred to poly(styrene-alt-maleic anhydride) (PSMA) coated coverslips allowing covalent immobilization of collagen to the glass substrate. Topological parameters of the reconstituted matrices, namely, pore size and fibril size, were analyzed using a custom-built image processing toolbox as previously reported [42]. Then, porosity was calculated as the ratio of the void volume to the total volume of the matrices.

#### 2.3.2. Permeability Analysis

A practical experimental setup (Figure S1) was developed to measure permeability of hydrogels. A hydrogel layer was placed in a 2 mL vial with a previously cut base. A nylon mesh (pore diameter = 70 μm) was incorporated at the bottom of the vial to hold the hydrogel in place. While the hydrogel is still in its liquid state, the mesh was covered with parafilm layers to avoid hydrogel leaking from the mesh. After hydrogel-crosslinking, an aperture was made in the cap of the vial through which a tube filled with distilled water was inserted. A scale (W3100A-120 Analytical Balance, Accuris Instruments, Edison, NJ, USA) was placed under the vial to measure the mass of the water droplets coming out of the hydrogel layer and the time instants at which each droplet fall was recorded. Then, permeability was calculated according to Darcy’s law:

k = (v∙μ∙Δx)/ΔP

where:

k is the permeability of the hydrogel (m^2^), v is the average fluid flow velocity through the hydrogel (m/s) calculated given the experimentally measured mass flow rate of the fluid’s Qmass (kg/s), fluid density ρ (kg/m^3^), and the cross-sectional area of hydrogel A (m^2^) as follows:

v = Qmass/(ρ∙A)

μ is the dynamic viscosity of the fluid (Pa∙s), Δx is the measured thickness of the hydrogel layer (m), ΔP is the applied pressure drop (Pa) calculated given fluid density ρ (kg/m^3^), gravitational acceleration g (m/s^2^), and fluid’s piezometric height Δh (m) as follows:

ΔP = ρ∙g∙Δh

#### 2.3.3. Hydrogel Mechanics

Matrix elasticity was quantified using ElastoSense Bio2 (Rheolution, Montreal, QC, Canada). 3 mL of the prepared collagen solution were transferred to the sample holder and the fibrillation was initiated at 37 °C.

### 2.4. Flow Simulation

The CAD file describing the geometry of the device was imported in a platform for the finite element method (FEM) analysis (Comsol Multiphysics ver. 5.2, COMSOL, Stockholm, Sweden) to evaluate pressure and fluid velocity in the device.

A stationary analysis was performed considering the following boundary conditions: reference pressure at the outlet (0 mbar); flow rate of 0.01 mL/min at the inlet; no-slip conditions on the remaining fluid-solid interface. The infused fluid was assumed to be pure water. The region occupied by the hydrogel was assigned the porosity and permeability values obtained from hydrogel characterization (Section 3.1).

Fluid flow is modelled using the Navier–Stokes equations in the region of free pressure-driven flow, while the flow through the porous hydrogel is modelled according to the Brinkman equations, which extend Darcy’s law to account for energy dissipation due to shear stresses [43,44].

### 2.5. Perfusion of the System

The device was connected to microtubing at the inlet and outlet, filled with sterile water, and sealed using the clamping system. The device was then connected to a micropump (PHD Ultra, Harvard Apparatus, Holliston, MA, USA) at the inlet, which was adjusted to pump a water solution colored with a trypan blue dye (Gibco, Fisher Scientific, Waltham, MA, USA) into the device at a controlled flow rate. The flow of the colored solution within the device was then monitored using live imaging microscopy (Zeiss AxioCam HRm, Carl Zeiss, Oberkochen, Germany). The flow of the colored solution was similarly observed through collagen hydrogels previously described, which were loaded into the compartments of the LN-on-a-chip.

### 2.6. Compartmentalization Assessment

In order to demonstrate efficiency of micropillars in maintaining zonal distribution, collagen hydrogels with varying colors were prepared and successively loaded into the compartments of the device. The first hydrogel, prepared as previously reported (Section 2.2), was loaded into region 1 and was polymerized for 30 min at 37 °C in a 5% CO_2_ incubator. The same steps were followed to load hydrogels in region 2, region 3A, and finally region 3B. Gels were then left in the incubator for an additional hour for complete gel polymerization before the device was transferred onto the microscope stage for imaging.

### 2.7. Cell Culture

Human EB1 (Cell Lines Service, Eppelheim, Germany), THP-1 (ATCC, Manassas, VA, USA), and Jurkat cells (ATCC, USA) were grown in a Roswell Park Memorial Institute (RPMI) 1640 medium containing d-glucose, 4-(2-hydroxyethyl)-1-piperazineethanesulfonic acid (HEPES), l-glutamine, sodium bicarbonate, and sodium pyruvate (Gibco) supplemented with 10% fetal bovine serum (FBS) (Gibco) and 1% penicillin–streptomycin (Biosera, Nuaille, France). Mature dendritic cells (DCs) were differentiated from THP-1 cells as previously reported [45]. The cells were cultivated in an RPMI 1640 medium supplemented with 100 ng/mL Granulocyte-macrophage colony-stimulating factor (GM-CSF), 200 ng/mL Interleukin-4 (IL-4), 20 ng/mL Tumor Necrosis Factor-Alpha (TNF-α), 200 ng/mL ionomycin. All cytokines and media supplements were supplied by BioLegend (San Diego, CA, USA). The differentiation was performed at standard cell culture conditions at 37 °C, 5% CO_2_, and 95% humidity for 3 days. Afterwards, the differentiated cells were detached using a TrypLE cell detachment solution (Thermo Fisher Scientific, Waltham, MA, USA). All immune cell types were then mixed with prepared collagen solutions and loaded into the LN-on-a-chip in their corresponding zones.

To distinguish between the different immune cell types within the device, CellTracker (Invitrogen, Thermo Fischer Scientific, Waltham, MA, USA) dyes were used for short-term labelling of the cells following the manufacturer’s protocol. In brief, the CellTracker dye stock was prepared at 0.01 M in 0.22 mm sterile-filtered Dimethyl sulfoxide (DMSO) (Sigma, St. Louis, MO, USA). A staining working solution was then prepared by diluting the stock to 1 mM in a serum-free medium. The cells were suspended in the staining solution, incubated for 30 min at 37 °C and 5% CO_2_, and eventually resuspended in a full growth medium and used for experiments within 1–3 days.

### 2.8. Device Cytocompatibility 

The ultimate goal of the developed LN-on-a-chip is to enable studies aimed at quantifying immune responses and examining cell–cell interactions as well as cell–antigen interactions. Hence, assessing compatibility of the device with human immune cells is key to ensure that the chip itself, or the process of culturing the cells in the system, has no significant effect on cell viability and overall health. First, an initial viability test was conducted for three common immune cell lines (EB1, THP-1, and Jurkat) cultured in the LN-on-a-chip system under static conditions for 24 h. Next, extended viability tests of 2 cell types cultured in the LN-on-a-chip were conducted for 72 h under both dynamic and static conditions. Continuous perfusion of cell media into the device was maintained over the 72-h period using a microfluidic pump (PHD Ultra, Harvard Apparatus, Holliston, MA, USA) and microwell plates were used as a control. After incubation, the cell-containing hydrogels were retrieved from the device by disassembling the mechanical clamping system and removing the hydrogel via pipetting. Collagen hydrogel was then digested with 2 mg/mL collagenase (Merck, Kenilworth, NJ, USA) prepared in an RPMI 1640 medium in order to isolate the cells. Once isolated, the cells were assessed for viability using 7-aminoactinomycin D (7AAD) (Biolegend, San Diego, CA, USA). This fluorescent DNA dye is used to detect apoptosis in flow cytometry studies. Guanine-specific synthetic fluorescent analogue of actinomycin D intercalates DNA by binding to guanine-cytosine (GC) regions, thus discriminating between live, early, and late apoptotic cells in flow cytometry, with excitation/emission = 488/647 nm. Briefly, samples were resuspended in the cell staining buffer (Biolegend, USA) stained with 7AAD at a ratio of 1:50 (*v*/*v*), incubated for 30 min at 4 °C in the dark, and finally washed and resuspended in the washing buffer for analysis with a BD Accuri C6 flow cytometer (BD Biosciences, Franklin Lakes, NJ, USA). Two different controls cultured in commercially available well plates were used to determine 7AAD staining specificity: a live cells control and a dead cells control, which was prepared by boiling cells at 100 °C for 30 min. All flow cytometry results were analyzed using the software of the same cytometer.

To assess functionality of the cells within the LN system, a cell proliferation assay was performed. Cell proliferation was determined using a CellTrace^TM^ Carboxyfluorescein succinimidyl ester (CFSE) Cell Proliferation Kit (Biolegend, USA). Jurkat T cells were incubated with 1 µM CSFE dyes prepared in an RPMI 1690 medium for 15 min at 37 °C, 5% CO_2,_ and 95% humidity. To remove the CSFE dye, the cells were centrifuged and the cell pellet was resuspended in the prepared collagen solution. The collagen solution was injected into the LN-on-a-chip and cultured with and without the presence of matured DCs derived from THP-1 cells for 3 days in static or flow conditions. Continuous perfusion of cell media into the device was maintained over the 72-h period using a microfluidic pump (PHD Ultra, Harvard Apparatus, Holliston, MA, USA) and microwell plates were used as a control. The flow rate was set to 1 µL/min. To analyze the T cell proliferation, collagen matrices were manually separated from the LN-on-a-chip and digested with 2 mg/mL type IV collagenase prepared in an RPMI 1690 medium. The cells were analyzed using flow cytometry (Attune, Invitrogen, Carlsbad, CA, USA) regarding the CSFE fluorescent signal. The obtained results were quantified using computational software FLOWJO (BDFlowJo LLC, Ashland, OR, USA). For the quantitative analysis, the cell expansion index was used to determine the fold expansion of the overall culture.

### 2.9. Data Analysis

All the experiments were done at least in triplicates, and the data were presented as the means ± standard deviations.

## 3. Results

### 3.1. Collagen Matrix Characterization

**Figure 2:**
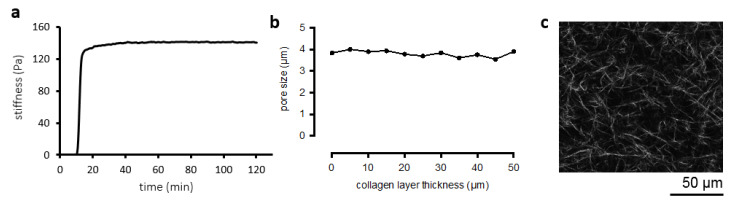
Collagen hydrogel characterization. (**a**) The stiffness (Young’s modulus) of collagen matrices versus time of hydrogel gelation. When the hydrogel is incubated at 37 °C, the hydrogel starts to polymerize, and the stiffness increases gradually until it reaches a plateau. (**b**) Pore size versus collagen layer thickness. The pore size is almost the same in all collagen layers indicating homogeneity of the hydrogel. (**c**) Confocal image of a fluorescently labelled collagen network.

Hydrogel characterization results using type I collagen concentrated at 2 mg/mL are reported in Figure 2. Collagen substrate architecture revealed fibrillar morphology (Figure 2c). The stiffness of the collagen matrix was 138.62 ± 1.54 Pa, within the range of the human LN stiffness, which is 120 Pa to 1 kPa as reported in [46] (Figure 2a). Collagen permeability, as measured with an in-house built experimental rig (Figure S1), was 3.84 × 10^−15^ ± 2.41 × 10^−16^ m^2^. Moreover, the porosity of the collagen network was found to be 74.35 ± 2.19% with the pore diameter of 3.973 ± 0.460 μm. Furthermore, our results also show homogeneity of matrices, illustrated by almost the same pore sizes across the samples (Figure 2b).

### 3.2. Flow Simulation

**Figure 3:**
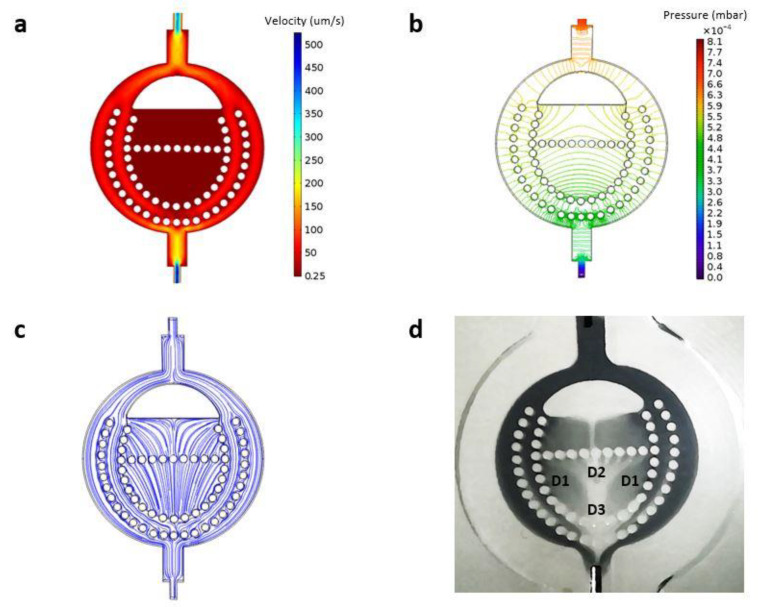
Flow within the LN-on-a-chip. (**a**) Magnitude of velocity field in the LN-on-a-chip. The highest velocities were at the inlet and the outlet. The lowest velocity was in the central area (regions 3A and 3B) with an average value of 0.25 µm/s. (**b**) Pressure distribution in the LN-on-a-chip (**c**) Streamlines showing direction of the flow within the LN-on-a-chip. (**d**) Flow of a colored water solution within the LN-on-a-chip. The flow gave rise to 3 functionally different districts within region 3B labelled D1, D2, and D3.

Figure 3a shows intensity of the kinetic field on the medial plane laying horizontal between the two bases of the device. For convenience, considering the order of magnitude of the involved physical quantities, in Figure 3, fluid velocity was expressed in µm/s, while pressure—in mbar.

Most of the afferent flow moved peripherally through regions 1 and 2, because these regions presented the least fluidic resistance to the incoming flow. The velocity of the fluid reached its highest values at the inlet and outlet cross-sections (peak velocity: about 0.6 mm/s). Figure 3b shows pressure (isobars) in the same plane. The pressure difference between the inlet and the outlet was about 8.1 × 10^−4^ mbar.

Interestingly, the central regions, namely, regions 3A and 3B, represent low-speed regions with an average velocity of 0.25 μm/s. The fluid arriving from region 2 entered region 3A and from there moved to region 3B, before discharging again into region 2 and, from there, to the outlet (Figure 3c)

The net flow through regions 3A and 3B was evidently zero, because the incoming flow must balance out the outgoing flow. The incoming (outgoing) flow through regions 3A and 3B can be computed as half the surface integral of the absolute value of the velocity flux calculated over the gel boundary. Such a flow rate (1.1 × 10^−9^ mL^3^/min) is several orders of magnitude smaller than the total flux through the device.

### 3.3. Flow Pattern within LN-on-a-Chip

To visualize direction of the fluid flow within the developed LN-on-a-chip which is dictated by the geometry of the chip, we utilized live imaging microscopy and observed distribution of a diluted trypan blue-colored water solution within the device. We observed that the inlet flow first arrived at regions 1 and 2 and then slowly moved into regions 3A and 3B. The fluid arriving at region 3A eventually moved into region 3B, after which the fluid from all the regions converged at the outlet and left the system (Video S1).

Interestingly, we also observed that the fluid flow towards region 3B gave rise to three functionally distinct districts within that same region. The different districts within region 3B are illustrated in Figure 3d. District 1 received inflow from region 2 as well as from region 3A. District 2 received inflow exclusively from region 3A. Finally, district 3 was an area of flow convergence from all the other districts. The flow distribution within region 3B is extremely important, especially for immunological studies and drug discovery applications, since region 3B represents the paracortex of the native LN housing T lymphocytes, which are critical determinants of immunological outcomes.

When the device was initially filled with hydrogels, the afferent flow followed a path similar to that seen with the device initially being filled with water but 300× slower. Most of the dye solution moved peripherally through region 1 and region 2, since they were the pathways of the least resistance. The fluid moved into regions 3A and 3B, but at a much slower rate than that seen when the device was initially filled with water at all the compartments.

### 3.4. Effect of Micropillars

**Figure 4:**
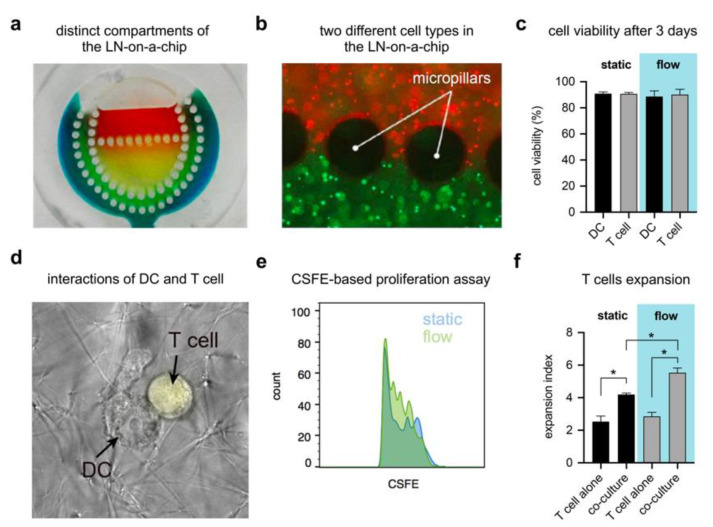
Biomimicry and cytocompatibility of the LN-on-a-chip. (**a**) Confinement of different hydrogels within distinct compartments of the LN-on-a-chip, which is facilitated by micropillars. (**b**) Proof-of-concept picture showing two different fluorescently labelled immune cells (Jurkat cells—red, THP-1—green), each in a distinct compartment within the LN-on-a-chip. (**c**) Cell viability of two different immune cell types, DC and Jurkat, cultured within the LN-on-a-chip for 72 h. The viability is over 90% for all the cell types. (**d**) Interaction between DC and Jurkat cells. Jurkat cells migrate across micropillar boundaries and seek DCs. (**e**) Carboxyfluorescein succinimidyl ester (CSFE) proliferation assay shows enhanced proliferation of cells with perfusion over 72 h. Proliferation of cells was higher in the LN-on-a-chip compared to conventional microwell plates. (**f**) T cells co-cultured with DCs show significantly enhanced proliferation, further augmented by media perfusion. Data are represented as mean ± SD; * significance level of *p* < 0.05.

To prove the efficiency of pillars in providing separation between the different compartments of the developed LN-on-a-chip, collagen hydrogels with varying colors were loaded successively into the compartments of the device before the whole device was sealed. Figure 4a shows that each of the hydrogels was confined in its associated compartment, which indicates that micropillars enable material confinement. Figure 4b clearly shows two different immune cells types (THP-1 and Jurkat cells) occupying distinct compartments within the LN-on-a-chip.

### 3.5. LN-on-a-Chip System Cytocompatibility

The cytocompatibility of the LN-on-a-chip system was assessed by culturing immune cells inside the device for 24 h and 72 h and then staining with 7AAD to discriminate apoptotic cells from live cells using flow cytometry. Our results show high viability rates within the LN system for both durations and for all cell types (Figure S2 and Figure 4c). The percentage of viable cells was 90% for both DC and Jurkat cells cultured in the perfused LN-on-a-chip, nearly identical to the cells cultured in microwell plates for the same duration (Figure 4c). The CSFE proliferation assay showed that co-culture of DC and T cells promoted enhanced T cell proliferation in both the control and LN-on-a-chip environments. Still, proliferation was further enhanced in the LN-on-a-chip (Figure 4e,f). Furthermore, we observed initial DC–T cell interactions within the perfused LN-on-a-chip (Figure 4d).

## 4. Discussion

Preclinical pharmaceutical screening still suffers from inaccuracies, as the pre-tested drugs ultimately encounter unexpected cytotoxic effects in humans. Recent evidence suggests that the immune system does play a role in drug toxicity. However, advanced platforms for precision screening are not available. Indeed, the complexity of such interactions among diverse cell types is not easy to be replicated with conventional cell cultures. Ideally, an LN-like environment could enable cell trafficking, zonal distribution, and interactions in a more reliable fashion than tissue culture flasks [7,47]. The aim of this study was to design and develop an LN microenvironment on a chip. The necessity of such a system stems from the lack of accurate in vitro platforms to assess immune response dynamics, including those pertaining to cell–cell interactions and cell–antigen interactions. As the microfluidic technology has emerged as an efficient means to reproduce key features of tissues or organs in vitro, we utilized it to develop an LN-on-a-chip that closely mimics the native human LN microenvironment. We showed that the developed LN-on-a-chip maintains cell viability for a duration of more than 72 h, which is sufficient for drug toxicity testing, and that it replicates the key features of the native LN, namely, the confinement of different cellular communities within distinct anatomical locations and the sustainment of a unique flow pattern. Furthermore, we showed that the collagen hydrogels used to encapsulate cells in the system possess characteristics comparable to the ECM components of the native LN. We also demonstrated that T cell proliferation is enhanced by both DC co-culture and continuous perfusion of the system.

To obtain an LN-on-a-chip, a number of components have been developed, which make it quite advantageous with respect to 2D cell cultures: (1) a suitable scaffold and chip for cell entrapment and maintenance of viability in a microfluidic system; (2) appropriate fluidics; and (3) a biomimetic device to allow cell compartmentalization. The use of natural biomolecules of the ECM, namely, proteins and glycosaminoglycans, provides external cues recognized by the cells to generate a niche [48]. Since type I collagen is the most abundant protein in the ECM of the LN [23,24], it was chosen as a hydrogel scaffold used to encapsulate immune cells loaded into the developed LN-on-a-chip. The obtained structure had indeed a fibrillar morphology and porosity which resembles that of the human LN [49] and numerous studies proved that growing cells in 3D influences morphology, molecular gradients, signaling pathways, secretion of growth factors, and gene expression [50,51,52].

To validate the LN-on-a-chip against its natural counterpart, we extracted information pertaining to the morphology, fluidics, and functionality of the native human LN from in vivo experiments and numerical models in literature and compared it to the data obtained with the LN-on-a-chip. We anticipated here that the developed LN-on-a-chip is not only morphologically similar to the native LN, but also has a consistent fluidic behavior, has compartments similar to the native human LN, and allows for timescale cellular experiments that enable the test durations comparable to the natural counterpart.

The reconstructed microenvironment possesses the characteristics comparable to the ECM components of the native LN, particularly, in terms of composition, morphology, porosity, stiffness, and permeability [46,53,54]. The data obtained in our system showed that the permeability of 3D hydrogels was comparable to that reported for the native LN (3.3 × 10^−15^ m^2^/s) [53]. In addition, porosity and mechanical properties (e.g., stiffness 120–1000 Pa) were in the range of those reported for LNs [46,54]. Having obtained a scaffold that resembles the main histochemical and physical characteristics of the LN ensures that the validation experiments performed using the device are performed in a physiologically-relevant microenvironment. Furthermore, compartmentalization is a key characteristic of the native LN allowing it to accurately screen the interstitial fluid for foreign substances [55,56]. Accordingly, the developed LN model is a multi-compartmentalized bioreactor containing micropillars that provide physical separation between the different device compartments [57]. Obtaining compartmentalization is of vital importance, since one important feature of the native LN is that each immune cellular type, specifically, T and B lymphocytes, populates a distinct microdomain within the LN. These microdomains also have a unique ECM composition [58]. ECM components can be easily modulated using the LN-on-a-chip through the preparation of unique 3D hydrogels. Furthermore, in terms of fluidics, the fluid flow within the LN-on-a-chip device was shown to be mainly through the peripheral regions of the device, from which some of the fluid then moves into the interior regions (regions 3A and 3B, Figure 1b). This, in fact, is comparable to the flow within the native LN, in which most of the incoming lymph fluid (90%) moves through the SCS and only a small fraction is directed to the cellular regions [28,39,59,60]. The direction of mass transfer within the developed LN-on-a-chip dictated by forced flow is well-suited to facilitate immune cell–cell interactions and cell–antigen interactions in a manner that closely mimics that in vivo. Natively, B lymphocytes within the follicle region of the LN get activated by receiving soluble antigens or antigen-loaded macrophages from the SCS [61]. Once activated, B lymphocytes move to the border between the follicle and paracortex, where they present antigen-derived peptides to T helper cells [62]. These B cells then receive signals from the T helper cells to proliferate, undergo isotope switching, and generate different classes of antibodies [63]. The antibodies then leave the LN and migrate to the site of infection. On the other hand, T lymphocytes within the paracortex of the LN get activated by receiving antigen-presenting cells, mainly DCs, from the SCS through the conduit network [64,65]. Once activated, T lymphocytes differentiate into effector cells, either cytotoxic (CD8+) T cells or helper (CD4+) T cells, and memory cells [66,67]. The cytotoxic T cells and helper T cells then leave the LN and migrate to the site of infection. Such material transfer is mediated by the geometry and fluidics of the developed LN-on-a-chip device. Region 3A of the developed LN-on-a-chip receives mass input from region 2, which is analogous to the follicle of the native LN receiving antigens and antigen-loaded macrophages from the SCS (Figure 3). On the other hand, region 3B of the developed LN-on-a-chip is divided into 4 functionally distinct districts with each district receiving mass input from different regions. District 1 receives input from regions 2 and 3A, which is analogous to the paracortex of the native LN receiving antigen-presenting cells from the SCS and B cells from the follicle to assist in their full activation. District 2 receives input exclusively from region 3A, which is analogous to the paracortex receiving B cells from the follicle to assist in their activation. District 3 receives input from all the other districts, which is analogous to the final immunological outcome of the native LN. The direction of material transfer in any system is not solely directed by forced flow, but also by diffusion, which is a fundamental driving force. The collagen gels used in this study to encapsulate immune cells have been shown to significantly hinder molecular diffusion, having diffusion coefficients ranging from 2.0 × 10^−11^ m^2^/s to 3.6 × 10^−10^ m^2^/s [68,69]. We estimated the speed of diffusion within the collagen hydrogels using Fick’s first law and the specific dimensions of the developed LN-on-a-chip and found that the speed of diffusion is 3 to 4 orders lower than that of forced flow. This indicates that pressure-driven flow is a significant contributor to the material transfer towards the cellular regions of the developed LN-on-a-chip. In addition, the gel impedes molecular diffusion more than it does for convection which is extremely beneficial, since it gives time for the cellular components in those regions to recognize and interact with incoming molecules. In fact, it has been shown that B cells do not get stimulated instantly when encountering antigens. Instead, they accumulate antigens over time, suggesting multiple rounds of antigen acquisition [70]. We also demonstrated through simulation and experiments that regions 3A and 3B are zones where flow velocity is extremely low (Figure 3). This is consistent with the findings in literature, in which the flow velocity in the follicle and paracortex of the native LN has been shown to range from 0.02 to 0.6 µm/s [28,32,71,72]. Given the tiny dimensions of LN conduits, even the smallest velocities result in significant shear forces affecting behavior of cellular residents in this region by altering their morphology as well as organization and inducing cytokine secretion [32]. In addition, the extremely low velocity in regions 3A and 3B is highly desirable to ensure enough time for antigens and/or antigen-bearing cells to find lymphocytes and induce their activation. Indeed, the longer the antigens or antigen-bearing cells reside within the cellular regions, the higher the chances they have to encounter a lymphocyte [73,74,75]. Natively, naïve B and T cells extravasate into the LNs via high endothelial venules. Once in the LN, T and B lymphocytes randomly crawl along the conduit network to reach the paracortex and the follicle, respectively. The lymphocytes then remain in their specific microdomains for a duration of 10-21 h waiting for an antigen encounter [76,77]. Once an antigen is encountered, an immune response is initiated and cells consequently proliferate and migrate to the site of infection. The obtained findings corroborate feasibility of a biomimetic platform of a LN, so called LN-on-a-chip. As fabricated, the device retains major features of its natural counterpart. It is additionally cytocompatible and made transparent to allow observation under the microscope, therefore, also enabling real-time phenomena related to the immune cells interacting with new substances to be studied.

The ultimate goal of the developed LN-on-a-chip is to quantify immune responses in real time and examine cell–cell interactions as well as cell–antigen interactions, such as organoids, which is more reliable in advanced 3D systems than in 2D systems [78]. Further advancements using the LN-on-a-chip could pave the way towards predicting drug immunotoxicity more precisely and reliably, thus contributing to increased safety, lowered cost, and shorter cycles for drug development.

## 5. Conclusions

We developed a novel microfluidic device that replicates the spatial microenvironment of the human LN. It incorporated distinct compartments for 3D immune cell culture and mimicked the unique flow pattern found in the native LN, which is a key regulator of adaptive immunity. The LN-on-a-chip was fabricated using ultra-precision Computer Numerical Control (CNC) machining and is accompanied by a clamping system to facilitate the experimental setup. Being transparent, the system enabled live microscopic imaging, thus allowing real-time monitoring of immune cells in a physiologically-relevant microenvironment. The ultimate goal of the LN-on-a-chip is to provide a more accurate in vitro platform to study the effect of pharmaceutical drugs to downstream immunology, hence, contributing towards less laborious and more cost-effective drug development.

The future work will focus on the development of a technology that enables mass production of LN-on-a-chip, which will allow the use of and screening on multiple devices simultaneously. This, in turn, will pave the way towards achieving detailed observation of immune cell behavior within the LN-on-a-chip in response to antigens and/or pharmaceutical drugs and classification of different immune response patterns. ”Candidate technologies include photolithography, oxygen plasma bonding, and bioinspired hydrogels [79,80,81].”

## 6. Patents

The design of the LN-on-a-chip has been submitted for an international patent application by Khalifa University of Science and Technology (patent serial no. 16/650,218).

## Supplementary Materials

The following are available online at https://www.mdpi.com/1999-4923/12/5/464/s1: Figure S1. Schematic of the experimental setup for permeability analysis; Figure S2. Cell viability of three different immune cell types, THP-1, Jurkat, and EB1, cultured within the LN-on-a-chip for 24 h under static conditions; Video S1. Fluid flow within the developed LN-on-a-chip.
