# Supplementary figures and images for: Multi-Compartment 3D-Cultured Organ-on-a-Chip: Towards a Biomimetic Lymph Node for Drug Development
Source: Pharmaceutics. 2020 May 19;12(5):464. doi: 10.3390/pharmaceutics12050464 (PMC7284904; doi:10.3390/pharmaceutics12050464)

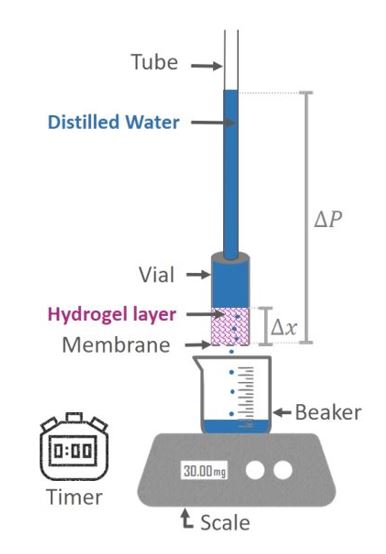

Supplement: Supplementary file 1 [file pharmaceutics-12-00464-s001.zip › Figure S1.JPG]

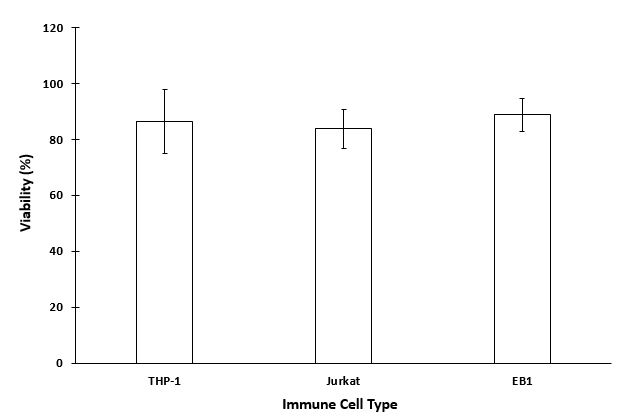

Supplement: Supplementary file 1 [file pharmaceutics-12-00464-s001.zip › Figure S2.JPG]
